# Design Rule of Mach-Zehnder Interferometer Sensors for Ultra-High Sensitivity

**DOI:** 10.3390/s20092640

**Published:** 2020-05-05

**Authors:** Yiwei Xie, Ming Zhang, Daoxin Dai

**Affiliations:** 1 Centre for Optical and Electromagnetic Research, State Key Laboratory for Modern Optical Instrumentation, College of Optical Science and Engineering, International Research Center for Advanced Photonics, Zhejiang University, Zijingang Campus, Hangzhou 310058, China; yiweixie@zju.edu.cn (Y.X.); mingzhang@zju.edu.cn (M.Z.); 2 Ningbo Research Institute, Zhejiang University, Ningbo 315100, China

**Keywords:** integrated optics, polarization-selective devices, Mach-Zehnder interferometer sensor

## Abstract

A design rule for a Mach-Zehnder interferometer (MZI) sensor is presented, allowing tunable sensitivity by appropriately choosing the MZI arm lengths according to the formula given in this paper. The present MZI sensor designed by this method can achieve an ultra-high sensitivity, which is much higher than any other traditional MZI sensors. An example is given with silicon-on-insulator (SOI) nanowires and the device sensitivity is as high as 10^6^ nm/refractive-index -unit (or even higher), by choosing the MZI arms appropriately. This makes it possible for one to realize a low-cost optical sensing system with a detection limit as high as 10^−6^ refractive-index-unit, even when a cheap optical spectrum analyzer with low-resolution (e.g., 1 nm) is used for the wavelength-shift measurement.

## 1. Introduction

The demand for label-free, low-cost, highly-sensitive and compact optical sensors keeps increasing rapidly in many areas, such as biological, environmental and chemical detections [1,2,3,4,5,6,7,8,9,10]. Integrated optical waveguide sensors have been developed as one of the best candidates to satisfy this demand [11,12]. Integrated optical waveguide sensors usually have a sensing window covered by the target analyte. When the concentration of the target analyte varies, the effective refractive index of the waveguide touching the target analyte changes, due to the evanescent field effect. Consequently, a phase-delay variation is introduced when light propagates along this optical waveguide. Some specific photonic circuits are then used to transfer this kind of phase variation to light intensity variation or wavelength shift, which could be measured by using a powermeter or an optical spectrum analyzer (OSA). Sensitivity improvement is desired for a sensor, so that a small variation could be detected with regular low-cost measurement instruments (e.g., low resolution OSA) [13].

It is well known that the sensitivity for an optical waveguide sensor includes two parts. One is the waveguide sensitivity *S*_1_, which is determined by the optical waveguide structure. Recently, silicon-on-insulator (SOI) nanowires have been widely used for optical sensing, because of the high sensitivity of the waveguide, which results from the enhancement of the evanescent field, due to submicron cross section and the ultra-high index contrast [1]. The other one is the device sensitivity *S*_2_, which is determined by the design of the photonic circuits. In this paper, we focus on the latter. There are two popular types of integrated optical waveguide sensors. One is based on optical microcavities, like the microring/microdisk [3,4,5,6,7], which have attracted much attention because of the ultra-compact size and large scale integration. Usually, an optical microcavity sensor is used by measuring the resonant wavelength shift Δλ, and the device sensitivity is defined as *S*_2_ ≡ Δλ/Δ*N*, where Δ*N* is the change of effective index. One can obtain *S*_2_ = λ_0_/*N*_g_ [14], where λ_0_ is the resonant wavelength and *N*_g_ is the group index of the microcavity waveguide. For a microcavity based on regular SOI nanowires, which has a group index of around 3~4, the device sensitivity is about 400~500 nm/RIU (refractive index unit). In order to detect a very small refractive index change, e.g., ∆*n*_s_ = 10^−6^, one has to develop an ultra-high Q micro-cavity and utilize an expensive and unwieldy high-resolution OSA (or tunable laser), so that the sub-picometer wavelength shift could be distinguished as required. In order to make the low-resolution OSA available for an integrated optical sensor, one option is to use the recently proposed optical sensor based on cascaded high-Q ring-resonators [15], which have ultra-high sensitivities of 10^3^~10^5^ nm/RIU, due to the Vernier effect [15,16,17,18,19,20,21]. For example, in [17], the demonstrated cascaded-ring optical sensor has a sensitivity of up to 4.6 × 10^5^ nm/RIU and a detection limit of 0.48 × 10^−5^ RIU in experiments. This idea has been extended for the cases with more than two rings recently [20,21]. However, the main resonance wavelength of the cascaded-ring resonator shifts in a digital way. Consequently, the detection resolution is not very high (∆n_s_~10^−5^).

The other popular type of integrated optical waveguide sensors is based on two-beam interference, such as Mach-Zehnder interferometers (MZI), featuring a broad dynamic range and long interaction length [22,23,24,25,26,27]. Usually, an MZI sensor is used by measuring the output power change for a given single wavelength. However, it is not an appropriate choice because [27]: (1) it is sensitive to the power variation of the light source; (2) the sensitivity decreases to zero at the extrema of the transmission curve; (3) the operation wavelength has to be tuned to achieve the optimal operation. In contrast, a frequency-resolved operation is an alternative to overcome the drawbacks of the single-wavelength method. Numerous efforts have been demonstrated to improve device sensitivity. An MZI sensor using silicon platform with hollow type hybrid plasmonic waveguide has been demonstrated, the device sensitivity *S_d_* is 157.4 nm/RIU for the MZI sensor, with a 20 μm hollow HP waveguide in the sensing arm [23]. In [24], a silicon photonic biosensor was demonstrated by using cascaded MZI and ring resonator with the Vernier effect. The achieved sensitivity is up to 21,500 nm/RIU [24], which is much higher than the sensitivity of 2870 nm/RIU for a regular MZI sensor. More recently, in [25], an integrated plasmo-photonic liquid refractive index sensor based on an MZI with amplitude and phase tuning elements has been realized, with a bulk sensitivity of 1930 nm/RIU experimentally. In addition, they also show the sensitivity may be up to 60,000 nm/RIU by engineering the free spectral range to be 600 nm. A SiN sensor chip consisting of an MZI and an arrayed-waveguide grating spectral analyzer was realized, with a detection limit of 0.6 × 10^−^^5^ RIU [26]. In Ref. [27], a regular MZI sensor with two equally length arms is discussed to achieve high sensitivity, which is given by *S*_2_ = λ_0_/(*N*_g1_−*N*_g2_). Here, the group indices (*N*_g1_, *N*_g2_) are dependent on the waveguide structure. Nevertheless, the sensitivity can still easily be much higher than that of a microcavity sensor. In this paper, we derive the formula for the sensitivity of an MZI sensor with asymmetric arms for the first time, and prove that it is possible to achieve an arbitrarily high sensitivity as desired, when the MZI arm lengths are appropriately chosen, according to the derived formula. An example is given with SOI nanowires, and the sensitivity is as high as 10^6^ nm/RIU (or even higher). Such ultra-high sensitivity makes it possible to realize a low-cost optical sensing system with a detection limit as high as 10^−6^ RIU, even when using a cheap optical spectrum analyzer with a low high-resolution (e.g., 1 nm).

## 2. Principle

**Figure 1:**
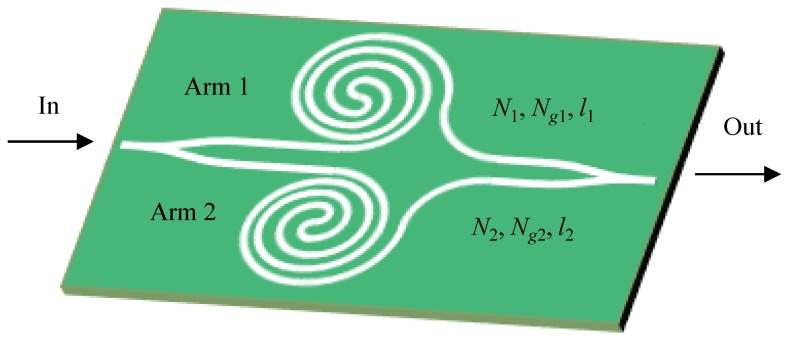
A Mach-Zehnder interferometer (MZI) sensor with optimal arm-lengths (*l_1_, l*_2_). Here, the arm lengths are given as *l*_2_ = (*m*+1/2)[*N*_1_−(*D*_1_λ_0_+ε)]/[*N*_2_(*D*_1_+ε/λ_0_)− *N*_1_*D*_2_], and *l*_1_ = *l*_2_ *N*_g2_/(*N*_g1_ − ε).

Figure 1 shows the schematic of an MZI sensor with two unequally long arms, which can usually be spiraled to be compact [1]. For an MZI, one has the following equation

*N*_1_, *N_g_*_1_, *l*_1_ − *N*_2_*l*_2_ = (*m* + 1/2) λ_0_ (1)

where *N_i_* and *l_i_* are the effective index and the length of the *i*-th MZI arm waveguide (*i* = 1, 2), respectively, *m* is the interference order, and λ_0_ is the central wavelength. When the analyte covered on an arm waveguide has a refractive index change of Δ*n*_s_, the corresponding variation Δ*N*_1_ of the effective index is given by

Δ*N*_1_ = Δ*n*_s_ (∂*N*_1_/∂*n*_s_) ≡ Δ*n*_s_*S*_1_ (2)

where *S*_1_ is the so-called waveguide sensitivity [14,28]. The central wavelength of the MZI then becomes λ_0_+Δλ and one has

(*N*_1_ + *D*_1·_Δλ + Δ*N*_1_)*l*_1_ − (*N*_2_ + *D*_2_Δλ)*l*_2_ = (*m* + 1/2) (λ_0_ + Δλ) (3)

where *D*_1_ = ∂*n*_eff1_/∂λ and *D*_2_ = ∂*n*_eff2_/∂λ are the dispersion coefficients of the first and second MZI arm waveguides, respectively.

According to Equations (1) and (3), the device sensitivity *S*_2_ of an MZI sensor is given as follows:

*S*_2_ ≡ Δλ/Δ*N*_1_ = λ_0_/(*N*_g1_ − *N*_g2_·*l*_2_*/l*_1_) (4)

where the group index *N*_g*i*_ =*N_i_*−λ_0_∂*N_i_*/∂λ, *i* =1, 2. The overall sensitivity of an MZI sensor is given by

*S* ≡ Δλ/Δ*n*_s_ = *S*_1_*S*_2_ (5)

In order to enhance the overall sensitivity *S*, one should maximize *S*_1_ as well as *S*_2_. High waveguide sensitivity *S*_1_ can be achieved by using nanophotonic waveguides with enhanced evanescent fields, such as SOI nanowires [14], nano-slot waveguides [5], and suspended waveguides [29,30]. In this paper, we focus on improving device sensitivity, *S*_2_, by optimizing MZI design.

According to Equation (4), it can be seen that an ultrahigh sensitivity *S*_2_ can be achieved by minimizing the denominator. Therefore, one expects to have the following equation

*S*_2_ = λ_0_/(*N*_g1_ − *N*_g2 *l*2_*/l*_1_) ≡ λ_0_/*ε* (6)

where *ε*<<1, and one has

*ε* ≡ (*N*_g1_ − *N*_g2 *l*2_*/l*_1_) (7)

Obviously, a smaller *ε* indicates higher device sensitivity (*S*_2_ = λ_0_/ε). It is possible to achieve any desired value to be *ε* << 1 by appropriately choosing the waveguide parameters (like *N*_g1_, *N*_g2_), as well as the lengths (*l*_1_, and *l*_2_), of the MZI arms. For a regular MZI sensor, usually one chooses *l*_1_ = *l*_2_ [27], and consequently the device sensitivity is determined by the difference of the MZI waveguides’ group indices, as given by *S*_2_ = λ_0_/(*N*_g1_ − *N*_g2_). When an ultra-high sensitivity is desired for a regular MZI sensor, one has to optimize the waveguide dimension (e.g., the core width) carefully, so that the difference (*N*_g1_ − *N*_g2_) is minimized to approach zero, which is possible. However, the optimal design is rather complicated, because the two MZI arm waveguides have different upper-claddings. It also makes the design inflexible for modifications of the optical waveguides for some specific considerations or applications.

In contrast, it is much easier and more flexible to adjust the lengths (*l*_1_, and *l*_2_) of the MZI arms, which is the method considered in this paper. Combining Equation (6) with Equation (1), the lengths (*l*_1_, *l*_2_) for the MZI’s arms are determined by the following formulas.

*l*_2_ = (*m*+1/2)[*N*_1_−(*D*_1_λ_0_+ε)]/[*N*_2_(*D*_1_+ε/λ_0_) − *N*_1_*D*_2_] (8)

*l*_1_ = *l*_2_*N*_g2_/(*N*_g1_ − ε) (9)

In this paper, we consider the design with SOI nanowires, which have commonly been used for optical sensing. The SOI nanowire used here has the following parameters: the core height *h*_Si_ = 220 nm, the core width *w*_co_ = 500 nm. The refractive indices for the silicon layer and the SiO_2_ layer are *n*_Si_ = 3.47644 and *n*_SiO_2__ = 1.44402, respectively. For the MZI arm with a sensing window, the upper-cladding is the liquid solution to be tested and the refractive index of the upper-cladding is *n*_cl1_ = 1.33. For the MZI arm waveguides with a SiO_2_ upper-cladding and liquid solution, the effective indices of the TM polarization fundamental mode are 1.7996 and 1.8501, respectively. The corresponding dispersion coefficients and group indices are (*D*_1_, *N*_g1_) = (−1.234 μm^−1^, 3.7123) and (*D*_2_, *N*_g2_) = (−1.182 μm^−1^, 3.9772). Here, TM mode is considered to achieve higher waveguide sensitivity. The center wavelength is *λ*_0_ = 1550 nm.

## 3. Results

**Figure 2:**
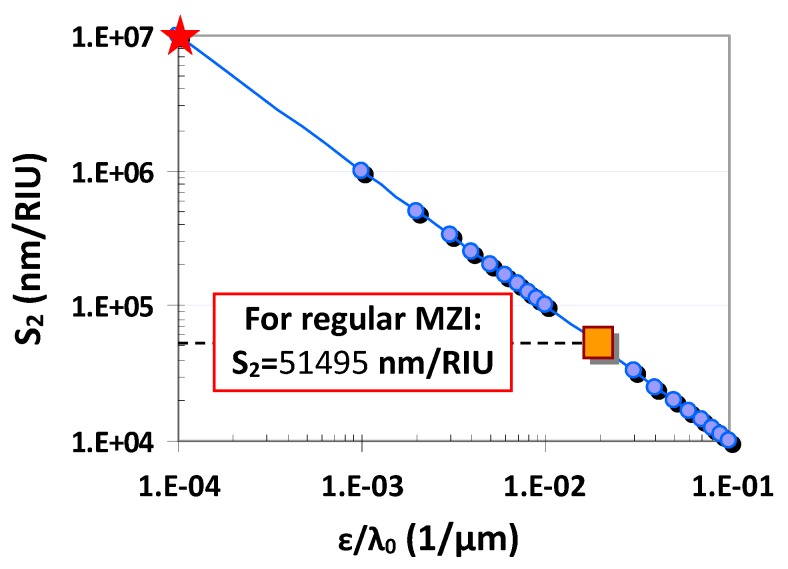
The device sensitivity *S*_2_ as the value ε/λ_0_ varies.

Figure 2 shows the calculated device sensitivity *S*_2_ as the value *ε/*λ_0_ varies. Theoretically speaking, one can realize an arbitrarily high sensitivity when making the value *ε* approach zero, which can be realized easily by choosing the MZI arm lengths according to Equations (7) and (8). For example, one has *S*_2_ = 10^7^ nm/RIU when ε/λ_0_ = 0.0001 μm^−1^. As a comparison, we also calculate the devices sensitivities for a regular MZI sensor with two equally long arms (i.e., *l*_1_ = *l*_2_), which is about 51,495 nm/RIU (see Figure 2). One can see that the present asymmetric MZI sensor enables much higher device sensitivity than a regular MZI sensor with *l*_1_ = *l*_2_.

Here, we give an example of designing an MZI sensor for a desired ultrahigh sensitivity (e.g., *S*_2_ = 10^6^ nm/RIU and ε/λ_0_ = 0.001 μm^−1^). One can obtain a solution for the lengths (*l*_1_ and *l*_2_) of the MZI arms by using Equations (7) and (8) for any given interference order *m*. For the case of ε/λ_0_ = 0.001μm^−1^ considered here, one has *N*_2_(*D*_1_+ε/λ_0_)−*N*_1_*D*_2_ < 0, and consequently, the interference order *m* should be an negative integer, so that the solutions for the lengths are positive, i.e., *l*_1_ > 0 and *l*_2_ > 0.

**Figure 3:**
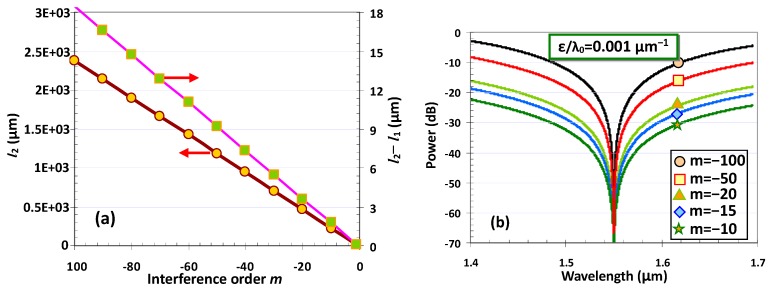
(**a**) The MZI arm length *l*_2_ and the length difference (*l*_2_−*l*_1_) when choosing different interference order *m*; (**b**) the calculated spectral responses of the MZI with different interference order *m*.

Figure 3a shows the calculated length *l*_2_ of the MZI arm and the length difference (*l*_1_−*l*_2_) by choosing the interference orders *m* in the range −100 to −1. It can be seen that a longer MZI arm is required when choosing a higher order *m*. Figure 3b shows the calculated spectral responses of the designed MZI with different interference orders *m*. The spectral responses are calculated with the formula of *T* = 10lg_10_[cos^2^(∆φ/2)], where ∆*φ* is the phase difference between the two MZI arms. It can be seen that there is a notch at the designed center wavelength 1550 nm, as expected. A higher interference order *m* makes a narrower notch, which is helpful in order to distinguish a smaller wavelength shift, due to the refractive index change. Fortunately, a small wavelength shift can be distinguished easily, even when choosing a moderate interference order *m*. Furthermore, a small interference order *m* is preferred to have short MZI arms, so that the MZI sensor becomes compact.

**Figure 4:**
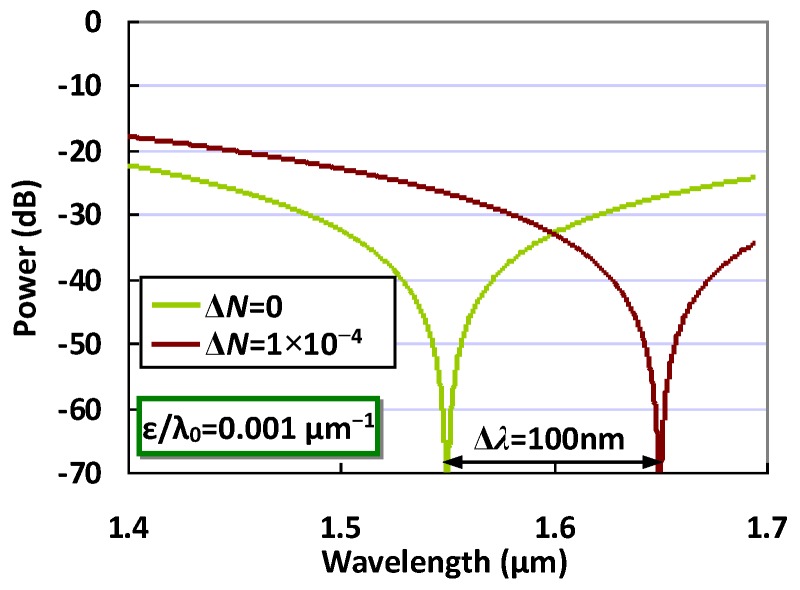
The calculated spectral responses of an MZI sensor when choosing ε/λ_0_ = 0.001 μm^−1^.

Therefore, in the following calculation, we choose *m* = −10, and the corresponding lengths of the MZI arms are *l*_1_ = 227.08μm and *l*_2_ = 228.84 μm. Figure 4 shows the calculated spectral responses of the designed MZI sensors when the effective index of the MZI arm waveguide has a variation of Δ*N*_1_ = 0 and 1 × 10^−4^, respectively. It can be seen that the notch wavelength shifts from 1550 nm to 1650nm. The wavelength shift Δλ is as large as 100 nm and the corresponding device sensitivity *S*_2_ = Δλ/Δ*N* = 10^6^nm/RIU. In this way, the detection limit for the refractive index change is as high as 10^−6^ RIU, even when using an OSA with a low resolution of ~1 nm. It can be seen that the MZI sensor with such an ultra-high sensitivity is beneficial, because one does not need a high-resolution OSA for the measurement of the spectral responses. An on-chop spectrometer, which usually has a relatively low resolution, will be available. This becomes very attractive for realizing a low-cost optical sensing system. In theory, it is even possible to further achieve a sensitivity as high as e.g., *S*_2_ = 10^7^ nm/RIU, by designing the MZI arm lengths appropriately when needed. In practical terms, one should realize that the sensitivity of the MZI sensor will be limited, due to some non-idealities. For example, when the powers split to the two MZI arms are not identical, the extinction ratio of the notch will degrade. In this case, there is no sharp notch, and thus a small wavelength shift might not be resolved. Fortunately, it is possible to achieve very uniform 1 × 2 3-dB power splitters on silicon by using mature symmetric Y-branches [31], or adiabatic tapered silicon waveguides [32]. Another key issue is that the lengths of the MZI arms usually have some random deviation from the design values due to the fabrication errors, and thus the value |*ε*| will increase to break the condition |*ε*| << 1. A possible solution is to introduce a thermally tuned phase-shifter to compensate the random variation of the lengths *l*_1_ and *l*_2_.

## 4. Conclusions

In summary, we have presented a novel design rule for achieving an MZI sensor with ultra-high sensitivity. For any given optical waveguide system, we have shown that tunable sensitivity can be achieved for an MZI sensor by choosing the MZI arm lengths appropriately. An example has been given with SOI nanowires, and the device sensitivity can be as high as 10^6^ nm/RIU (or higher). With such an MZI sensor, it is possible to detect a very tiny refractive index change as small as 10^−6^ RIU, even by using an OSA with a low resolution of ~1 nm. This is really helpful because a moderate/low-resolution OSA can be available for the measurement of the spectral responses. In particular, it is even possible to integrate with an on-chop spectrometer, which usually has a relatively low resolution. In this way, it becomes very attractive for realizing a low-cost optical sensing system.
